# Influence of natural and anthropogenic controls on runoff in the Keriya River, central Tarim Basin, China

**DOI:** 10.1371/journal.pone.0269132

**Published:** 2022-05-27

**Authors:** Jinhua Wang, Feng Zhang, Guangming Luo, Yuchuan Guo, Jianghua Zheng, Shixin Wu, Qalibinur Keram, Suhong Liu, Qingdong Shi

**Affiliations:** 1 College of Geography and Remote sensing Sciences, Xinjiang University, Urumqi, China; 2 Hotan Hydrology and Water Resource Survey Bureau, Hotan, China; 3 State Key Laboratory of Desert and Oasis Ecology, Xinjiang Institute of Ecology and Geography, Chinese Academy of Sciences, Urumqi, China; 4 School of Geography, Beijing Normal University, Beijing, China; 5 Key Laboratory of Oasis Ecology Ministry of Education, Xinjiang University, Urumqi, China; Institute of Earth and Environment, Chinese Academy of Sciences, CHINA

## Abstract

The potential impact of natural factors on the runoff of intermittent rivers and ephemeral streams (IRES) has been largely ignored in the Tarim Basin, China. A representative example is the Keriya River. To quantify the long-term dynamic variations in lower reach surface runoff of IRES, river length, defined as the distance between a selected fix point along the perennial river segment to its dynamic, ephemeral end, was used as an indicator. Using a total of 272 remote sensing images, we digitized and measured the distance (river length) between the center of Yutian County and the river’s end point on each image, and then calculated monthly inter-annual and intra-annual variations in length of the lower Keriya River from 2000 to 2019. Hydrometeorological data were combined with descriptors of anthropogenic disturbances to assess the relative influence of natural factors and anthropogenic disturbances on lower reach river runoff. The results showed that intra-annual variations in river length fluctuated seasonally, with the minimum value occurring in June; two main peaks occurred in March and August. The minimum June value in river length was closely linked to an increase in agricultural water demand and a decrease in upper reach runoff. The August peak in river length was related to the peak values in upper reach runoff and agricultural water demand; upper reach runoff made a significant contribution because the former was about 20% more than the latter in summer. The March peak corresponded to elevated lower reach groundwater levels and to the melting of ice along river channels. Inter-annual variations in river length were due to inter-annual variations in upper reach runoff and middle reach agricultural water use which increased slightly during the study period. Inter-annual variations in frequency and amplitude of the fluctuations in river length were mainly controlled by changes in upper reach runoff. The minimum in river length in 2009 was consistent with the low in upper reach runoff of the Keriya River and other rivers in the Tarim Basin. The most significant factors controlling variations in river length are natural in origin.

## 1. Introduction

Drylands encompass areas where the climate is classified as dry subhumid, semiarid, arid or hyper-arid; they cover about 41% of Earth’s terrestrial surface, and feed more than 38% of the global population [1]. They are also one of the most ecological fragile areas on the planet [2, 3] and contain intermittent rivers and ephemeral streams (IRES). IRES are defined as streams that cease to flow at some point along the river’s lower (downstream) reach [4]; they provide important ecological services that play a vital worldwide role in supporting biodiversity and human societies [5, 6]. IRES are often one of the most important sources of water in arid regions and constitute a large proportion of the total number and length of the planet’s rivers [7, 8]. The number and length of IRES are continuously increasing in global drylands as a result of climate change and human-induced water abstractions [4, 8]. Thus, the IRES of arid regions are becoming the focus of academic investigation. Nonetheless, most studies on rivers in arid regions have focused on perennial streams and rivers; there have been far fewer investigations of IRES [9]. The studies of IRES that have been conducted to date have mostly: (1) debated the definition of an IRES [4, 8, 10], (2) described their hydrological characteristics [7, 11–16], (3) developed conceptual models of stream flow [17, 18], (4) outlined the driving factors for their development [19–21], (5) assessed their hydrological connectivity [6, 22–24], and (6) discussed their management and protection [5, 9, 25–27]. The long-term change of IRES has not been strictly quantified on a global scale, and the universality, importance and fate of IRES have been neglected [6]. The limited study of IRES is partly due to the limited number of hydrological observation stations and limited monitoring data that are available [6]. An exception is the long-term and freely available time series data that can be obtained from remotely sensed images such as Landsat [28–32] and MODIS [33–35]. Both can provide effective supplemental ground observations. In fact, the analyses of these remote-sensing images provide a viable approach to quantify the long-term dynamic variations in runoff through IRES. For example, river length, defined as distance from a given datum to the end of the channel along its lower reach where water ceases to flow (and disappears), can be visually extracted from remote sensing images and used as a surrogate for surface runoff. Moreover, the data are relatively easy to obtain; only one remote sensing image is often needed for the digital measurement of river length per month. While the use of river length as documented herein may serve as a relatively effective approach, the number of studies specifically using this indicator to quantify runoff in IRES in arid regions remains small.

The Tarim Basin, located in an arid region in northwestern China, is the world’s largest inland arid basin, and possesses the planet’s second largest shifting sand desert [36, 37]. IRES are very common within the Tarim Basin [38]. During the past few decades, the basin has been affected by global climate change and intensified anthropogenic disturbances [39–41] that are associated with rapid population growth, large-scale land reclamation, and the mass exploitation of riverine surface water resources. As a result, water use and water shortages have become increasingly apparent [42]. Hence, the academic community has conducted numerous studies on the impact of human activities on runoff allocation and runoff change in the Tarim Basin [43–46]. Most rivers in the Tarim Basin represent IRES; thus, these recent investigations can be regarded as the study of IRES. Among them, many studies have argued that human activities in oases within the upper reach of the Tarim River are the main cause of decreased lower reach runoff [43–45, 47–56]. Admittedly, human activities have an influence on hydrological processes that have led to the lower reach reduction in surface runoff within IRES. Nonetheless, relatively little attention has been directed towards the analysis of natural hydrological processes on runoff, particularly the potential impacts of natural changes within the upper reaches of the basins flows on lower reach runoff [57].

**Fig 1:**
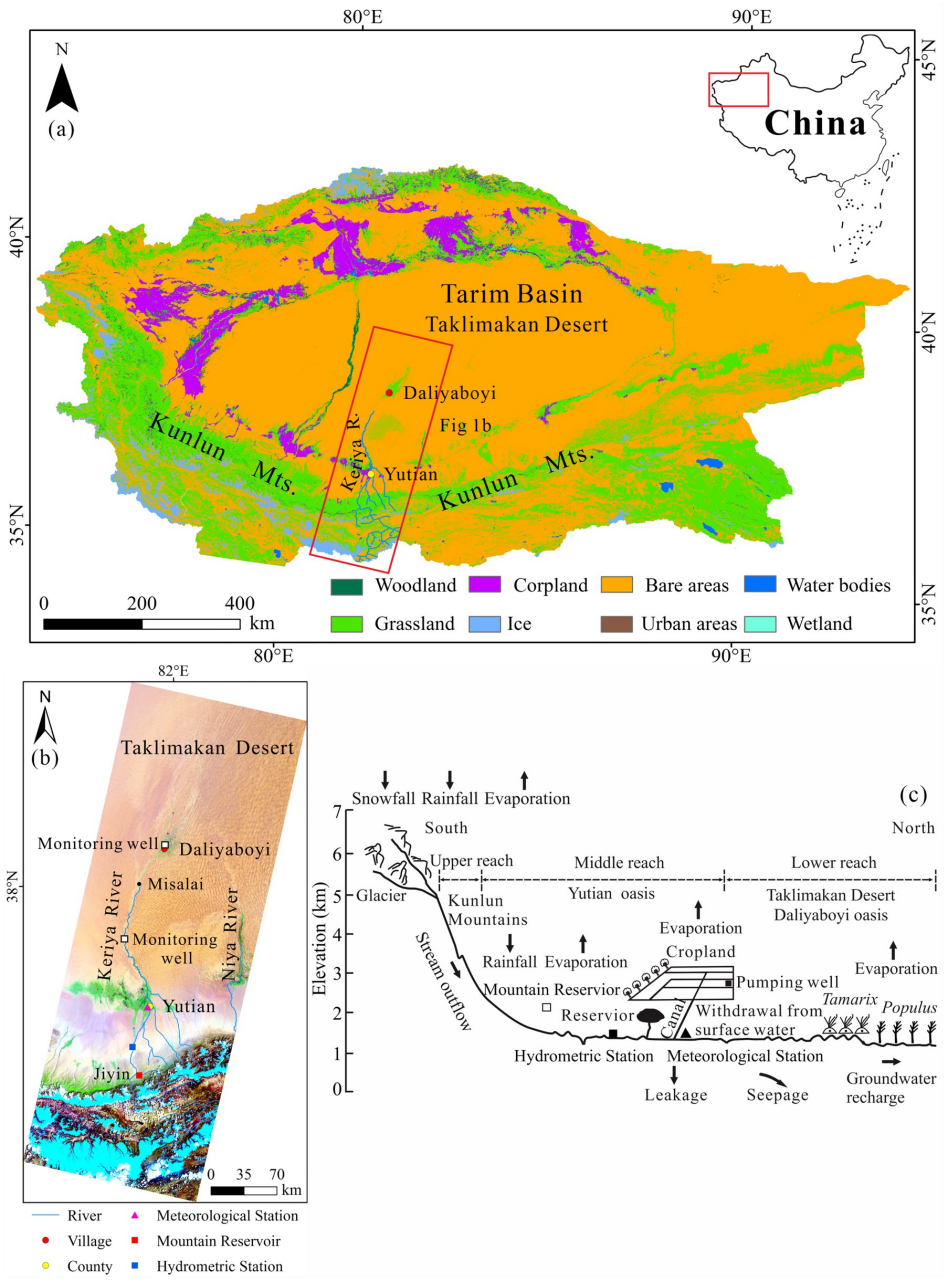
(a) Location of the Tarim Basin in China, and land cover in 2018 in the Tarim Basin. Land-cover map is available for download through the Copernicus Climate Change Service (C3S) Climate Data Store (CDS), at a spatial resolution of 300-m. The China map was downloaded from http://bzdt.ch.mnr.gov.cn/, and the grant number is GS(2020)3183; (b) The Landsat 8 OLI images based on a composite of Band 7 (Red), 5 (Green) and 2 (Blue). Images show the location of the Keriya River Basin. The river channel north of Misalai is ephemeral; south of Misalai the channel is perennial. (c) Conceptual model describing the controls on the variations in surface runoff, including factors within the mountains, the agricultural oasis, and the desert in Keriya River Basin. Land-cover map and Landsat image were downloaded from the website https://land.copernicus.eu/global/products/lc [2018], and http://landsat.visibleearth.nasa.gov/, respectively. Because the map and image downloaded from these websites are free and open to scholars, our study does not need to supply a copyright notice.

The Keriya River is an important seasonal tributary of the Tarim River [58]; it originates in the Kunlun Mountains (above 6,100 m a.s.l.) along the northern margin of the Tibetan Plateau [59] (Fig 1a). The Yutian agricultural oasis was developed along the middle reaches of the river [60] and is located where upper reach runoff flows out of the mountains (Fig 1a). Within downstream sections of the middle reaches of the river (used for human activities), the remaining water in the Keriya River flows into a natural oasis in the Daliyaboyi area (about 170 km away from the Yutian oasis). The Daliyaboyi area is located in the hinterland of the Taklimakan Desert [61] (Fig 1b). Variations in the length of the lower Keriya River can therefore be used to decipher the contributions of anthropogenic disturbances and natural variations in runoff on water availability and flow.

This study developed a long-term (2000–2019) time-series of the seasonal variations in river length from remotely sensed images of the lower reaches of the Keriya River. Combined with hydro-meteorological and other statistical data, the impacts of both natural and anthropogenic factors on the frequency and magnitude of temporal variations in runoff in the river basin were analyzed. This paper provides a new perspective for comprehensively understanding the impacts of anthropogenic disturbances and natural factors on hydrological changes along the lower reaches of the Keriya River. This study also advances and enriches our understanding of long-term variations in runoff within IRES in the Tarim Basin.

## 2. Materials and methods

### 2.1. Regional setting

The Keriya River has a length of about 740 km, including both a perennial and ephemeral stream reach [62]. The mean annual runoff in the upper Keriya River Basin is 7.57×10^8^ m^3^ (1958–2013) (provided by the Hotan Hydrology and Water Resource Survey Bureau of Xinjiang Uygur Autonomous Region, China). Runoff during the spring, summer, autumn and winter accounted for 11.7%, 66.4%, 14.7% and 7.2% of the total annual flow, respectively [63]. Annual flows are characterized by a single period of peak flow. The inter-annual variation in runoff of the Keriya River is limited (the coefficient of variation is 0.17) (1957–1984), even though its seasonal variation is significant [59]. The Keriya River Basin is divided into three general elevational zones: an upper zone of ice, snow and permafrost (altitude of 6100–2600 m a.s.l.); a zone dominated by an agricultural oasis in the middle reach of the basin (altitude of 2600–1400 m a.s.l.); and a zone characterized by desert along its lower reaches (altitude of 1400–1100 m a.s.l.) [62] (Fig 1c).

In the headwaters of the Keriya River Basin, there are 173 glaciers that cover a total area of 520 km^2^ and possess a total volume of 490×10^8^ m^3^ [63]. Seventy one percent of the Keriya River’s discharge is derived from the melting of glaciers and snow in its mountainous headwater regions; 9% is derived from precipitation, which is also mainly concentrated in the mountains. Groundwater contributes about 20% of the river’s flow [63]. The period of high (flood) flows within the upper reaches of the river is between July and August [59]. The mean annual precipitation is 129.2 mm in upper reach areas, while the mean annual evaporation between 1986–2013 was approximately 1902.4 mm (measured at the Langan Hydrological Station) (provided by the Hotan Hydrology and Water Resource Survey Bureau of Xinjiang Uygur Autonomous Region, China).

Within the zone possessing the middle reach agricultural oasis (approximately 1716 km^2^), the mean annual temperature is 11.8°C, the mean annual precipitation is 52.56 mm, and the mean relative humidity is 44.9% (calculated using the records from 1961 to 2019 at the middle reach meteorological station). The largest consumer of water is agriculture, which comprises about 98% of Yutian’s total water withdrawals; the combined use of water for industrial and domestic purposes represents only about 2% of the total [64]. The agricultural irrigation water is obtained from rivers, reservoirs and groundwater (springs and wells). Surface water bodies comprise about 86% (4.63×10^8^ m^3^ yr –^1^) of the total, whereas groundwater only accounts for 14% (0.74×10^8^ m^3^ yr –^1^) [64].

**Fig 2:**
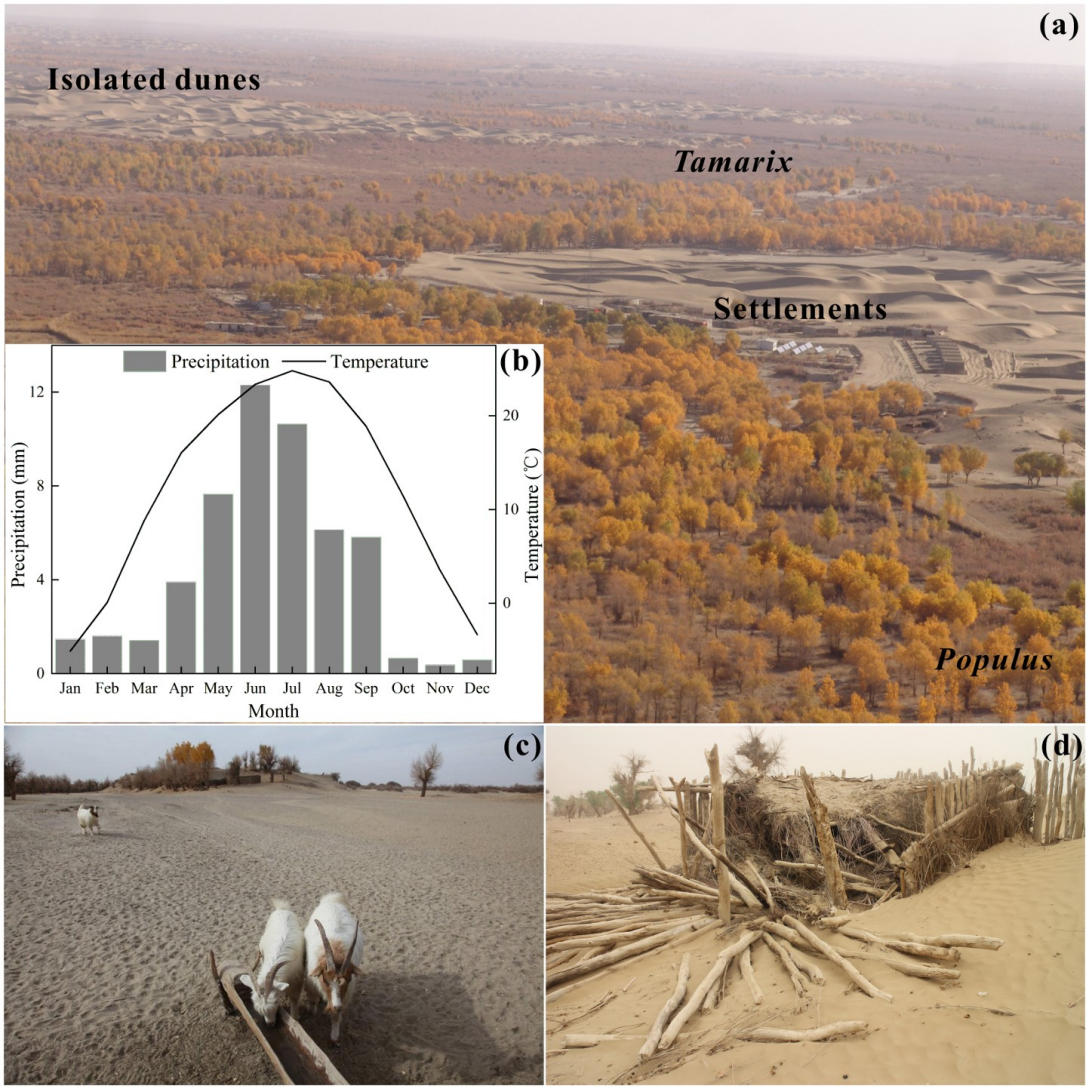
Photographs of Daliyaboyi natural oasis at the lower reaches of the Keriya River. (a) The vegetation (consisting of *Tamarix* and *Populus)* that surrounds the isolated dunes; the settlements are scattered in the delta; (b) Observed intra-annual variations in precipitation and temperature between 1961 and 2019 at the Yutian meteorological station located along the middle reach of Keriya River catchment; (c) Photograph of large-scale degradation and the general lack of desert riparian forests; (d) Photographs showing abandoned dwellings in the hinterland of the Taklimakan Desert. The study area is open to Chinese scholars for observation, and permits are not required to enter. Because these photographs were acquired during field observations by the authors of this article, the copyright belongs to this article.

The ephemeral river section of the Keriya River is located north of Misalai in most years [61] (Fig 1b). The flow to the oasis depends on the amount of surface water discharged from the middle reaches of the Keriya River. During the flood period, streamflow can traverse the ephemeral section and reach Daliyaboyi [60]. Daliyaboyi is the largest natural primitive desert oases within a present-day area and covers about 324 km^2^ [65] in the central Taklimakan Desert (Fig 1b). The oasis is maintained as a natural area [61]. There are no modern industrial and agricultural activities in the oasis. The mean annual temperature is about 11.8°C. The highest and the lowest temperatures occur in July and January, respectively [66]. The mean annual precipitation in the area is less than 10 mm; thus, the oasis is characterized by an extremely arid climate [62] (Fig 2b). Vegetation along the current riverbanks is comprised mainly of *Populus*, *Tamarix*, and *Phragmites* [67] (Fig 2a). The surrounding landscape is dominated by extensively isolated composite desert dunes, among which settlements are scattered [68]. The ecosystem of the oasis is extremely fragile [63] and covered by sparse vegetation that does not recover easily if destroyed. In recent decades, mid-to lower-reach regions have experienced severe competition in water demand [69] due to a significant increase in population and large-scale land reclamation in middle reach areas [70]. Decreased river discharge to Daliyaboyi, combined with declining groundwater levels, has had a particularly devastating, and possibly irreversible, impact on the lower reach natural oasis. The oasis has also experienced serious desertification [59, 65, 71–73] (Fig 2c and 2d).

### 2.2. Remote-sensing data collection

**Fig 3:**
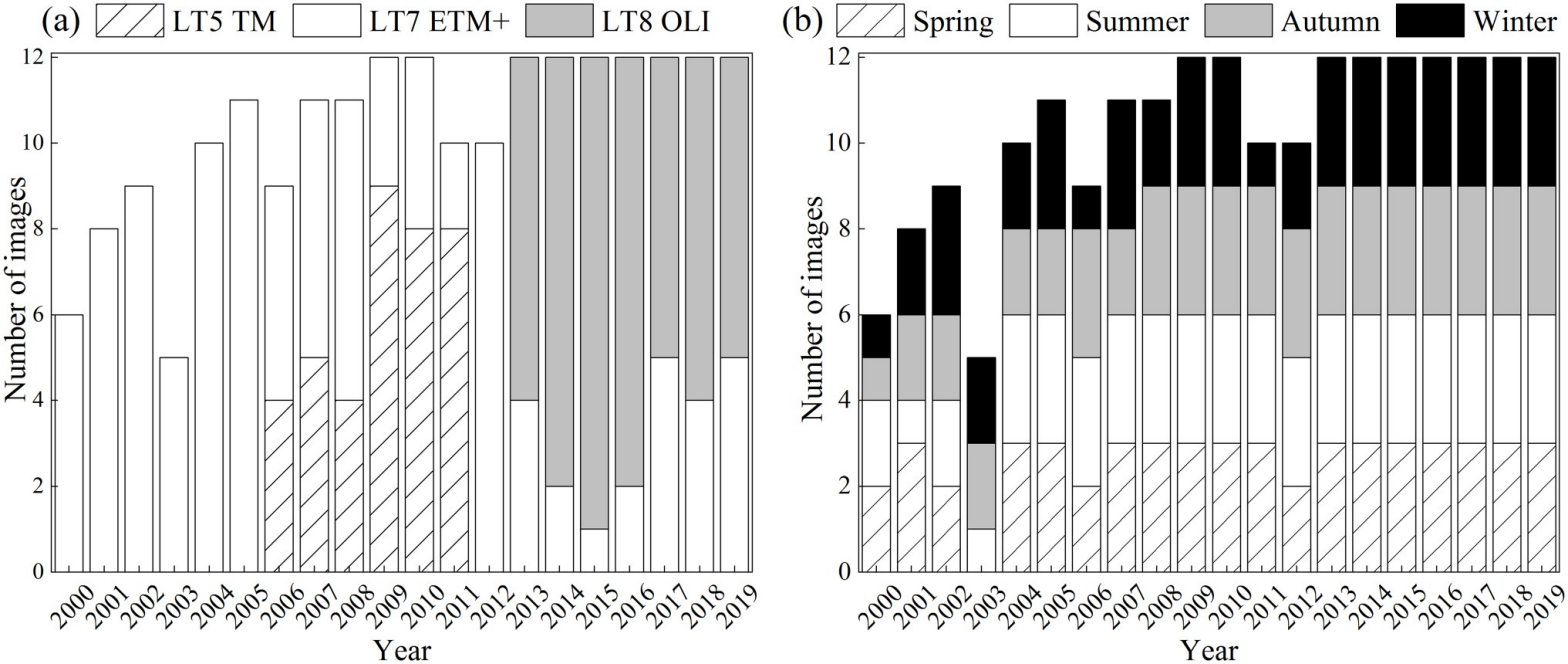
(a) Number of annual Landsat images. (a) by sensor type (Landsat 4–5 TM, Landsat 7 ETM+, and Landsat 8 OLI) and (b) by season (spring, summer, autumn, and winter) for the study area between 2000–2019. Note: the blank area in the figure represents missing Landsat images.

Remote sensing data (a total of 272 scenes) were obtained from Landsat images (240 scenes) (S1 Table), MODIS images (32 scenes), and Google Earth images (with a high-resolution of 0.47 m) (Table 1). More specifically, we used 45 Thematic Mapper (TM) images, 126 Enhanced Thematic Mapper Plus (ETM+) images, and 69 Operational Land Imager (OLI) images taken between January 1, 2000, and December 31, 2019. These images were obtained from the United States Geological Survey (USGS) (Fig 3). The study area is covered by two scenes (path/row 145/33 and 145/34) of the Landsat Worldwide Reference System (WRS-2). Landsat 5/7/8 Surface Reflectance (SR) products are standard level 1 Terrain-corrected (L1T) orthorectified Landsat images. A total of 32 Landsat images between 2000–2012 were unavailable. For these, we used MODIS images (MOD02QKM, Level 1B), which were obtained from NASA (National Aeronautics and Space Administration). The spatial resolution of MOD02QKM is 250 m. Thus, to cover the entire study area, one scene was required (horizontal/vertical 24/05), and these data were collected every 8 days.

**Table 1:** Summary of the remote-sensing data and data sources used in the analysis.

Data type	Year	Resolution	Data source
Landsat (TM, ETM+, OLI) Path/Row: 145/33, 145/34	2000–2019	30 m	http://landsat.visibleearth.nasa.gov/
MODIS (MOD02QKM) Tile ID: h24v05	2000–2012	250 m	http://earthobservatory.nasa.gov/
Global land cover map	2018	300 m	https://land.copernicus.eu/global/products/lc

### 2.3. Geometric correction

The mosaicked Landsat 8 OLI images of 26 April 2015 (path/row: 145/33 and 145/34) were first transformed to a common ground coordinate system (Universal Transverse Mercator (UTM), World Geodetic System (WGS 84) projection). The system is based on a 1:100,000 Standard Topographic Map of the People’s Republic of China and was created using the ENVI 5.1 image–processing software. Then we used a topographic map as the base image to geocorrect the mosaicked image (Landsat 8 OLI of 26 April 2015). Geocorrection used Ground Control Points (GCPs) of permanent features (such as settlements, dried channels, intersections of water bodies, and the interior of Daliyaboyi oasis) from the topographic map. The analysis used a second-order polynomial function with the nearest neighbor resampling method to produce an average Root Mean Square Error (RMSE) of less than 1 pixel [74, 75]. Approximately 100 GCPs were selected. The geometric corrected Landsat image (Landsat 8 OLI of 26 April 2015) was then used to rectify other Landsat images using GCPs (approximately 50–100). These images were also transformed using a second-order polynomial function and a nearest neighbor resampling method to produce an average RMSE of less than 1 pixel. The MODIS images were only used as a reference to distinguish the river end’s location on the corresponding geocorrected image (Landsat 8 OLI of 26 April 2015) for the river length calculation. Therefore, the MODIS images were not geometrically corrected.

### 2.4. Lower reach river length extraction, validation and statistical analysis

**Fig 4:**
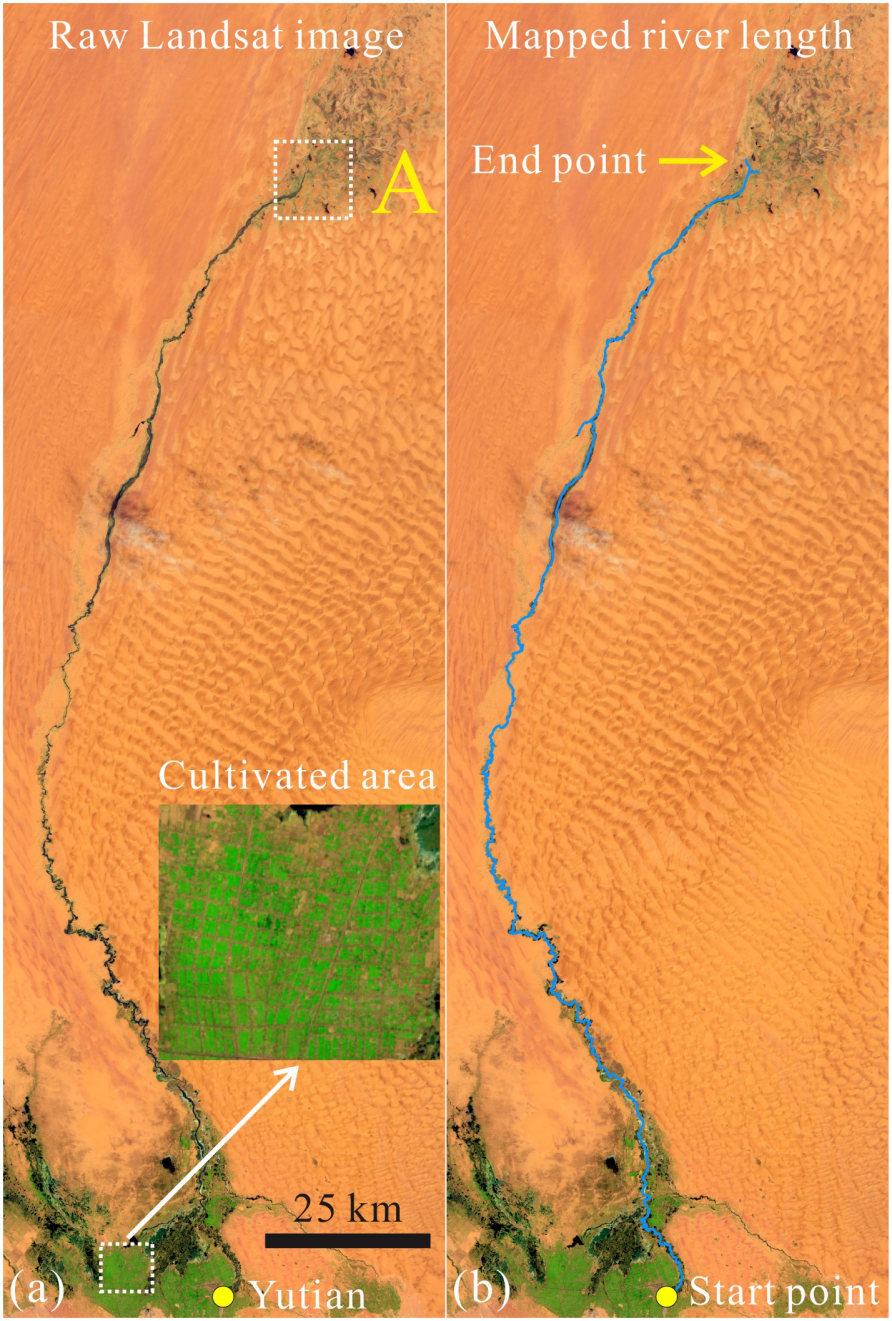
Measurement of river length and data validation process. (a) Raw Landsat images (pre-processed); (b) Extracted of river length between the center of Yutian County (yellow dot) and the river’s end (yellow arrow); the blue line represents mapped river length. Landsat image was downloaded from the website http://landsat.visibleearth.nasa.gov/. Because the images downloaded from this website are free and open to scholars, our study does not need to supply a copyright notice.

**Fig 5:**
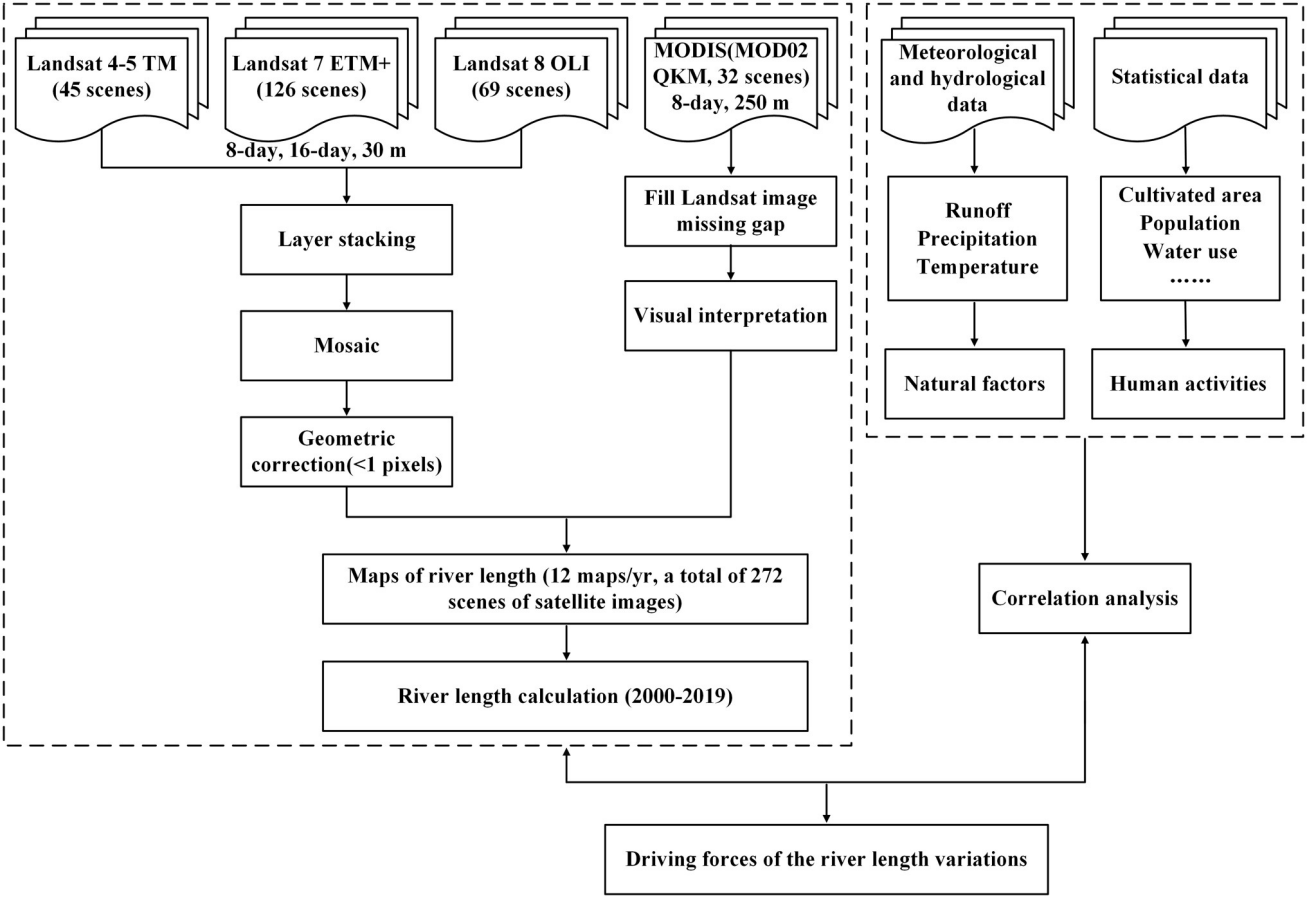
Schematic diagram of overall workflow, including processing of the remote-sensing data, the mapping and measurement of river length, and the analyses of the collected data.

The location of the river’s end point (site of flow termination) was visually identified on each Landsat image. The interpreted end points on the MODIS images were also marked on the geometric corrected Landsat images. The distance (river length) between the center of Yutian County (81°40’39" E, 36°51’20" N) and the river’s end point on each image (Fig 4a and 4b) was then digitized and measured using ArcGIS 10.1 (ESRI) software. We also used two high-resolution (0.47 m) Google Earth images captured on 26 August 2009 and 16 March 2016 to ensure our interpretations of the river end point were correct. The results were consistent with the Google Earth images. We also calculated statistical descriptors of the variations in river length, such as departure, accumulative departure, moving average, and coefficient of variation and correlation coefficient (Table 2), for the analyses (Fig 5).

**Table 2:** Description of the statistical indicators used in this study.

Indicators	Formula	Description
Departure	$x=x_{i}-\bar{x}$	*x*_*i*_ is the value in *i-*th year, and $\bar{x}$ is the average value during a specific period.
Accumulative departure	$LP_{i}=\sum_{i}^{N}\left(R_{i}-\bar{R}\right)$	*LP*_*i*_ is the cumulative anomaly in *i*-th year, *R*_*i*_ is the value in *i-*th year, $\bar{R}$ is the average value during a specific period.
Coefficient of variation	$CV=\frac{\mu }{\sigma }$	*CV* is the coefficient of variation, *μ* is the standard deviation, *σ* is the mean value.
Correlation coefficient	$r=\frac{\sigma_{xy}^{2}}{\sigma_{x}\sigma_{y}}=\frac{\frac{\sum \left(x-\bar{x}\right)\left(y-\bar{y}\right)}{n}}{\sqrt{\frac{\sum {\left(x-\bar{x}\right)}^{2}}{n}}\sqrt{\frac{\sum {\left(y-\bar{y}\right)}^{2}}{n}}}$	*r* is the Pearson product-moment correlation coefficient, $\sigma_{xy}^{2}$ is the covariance of variables *x* and *y*, and *σ*_*x*_ and *σ*_*y*_ is the standard deviation of *x* and *y*, respectively.
Moving average	$SMA=\frac{A_{1}+A_{2}+\cdots +A_{n}}{n}$	*SMA* is the simple moving average, where *A* is the average in period *n*; *n* is the number of time periods.

Due to the Landsat series of satellites having an 8-day or 16-day repeat cycle, up to three satellite images can be acquired for a given month but on different dates; although the longest interval between satellite images was 32-days, most images were taken at 16-day, 24-day intervals. Images with this temporal resolution are sufficient to capture the river length change in the lower part of the study area (Keriya River). Taking the data of Landsat imagery used in 2016 as an example (Table 3), the river length values digitized and measured from the three images in the same month are broadly continuous. In this paper, for those months with 2–3 image scenes, we used the mean value of river length; for the remaining months, one image scene was adopted. When both, one image and multiple scenes were used per month, actual variations in river length could be determined (Table 3). Therefore, our results of the visual interpretation and measurement of river length are reliable.

**Table 3:** Data information of Landsat imagery used and the results of river length in 2016.

Acquisition date	Sensor type	River length (km)
7-January-2016	Landsat 8 OLI/TIRS	315
31-January-2016	Landsat 7 ETM+	253
8-February-2016	Landsat 8 OLI/TIRS	315
16-February-2016	Landsat 7 ETM+	313
24-February-2016	Landsat 8 OLI/TIRS	323
3-March-2016	Landsat 7 ETM+	332
11-March-2016	Landsat 8 OLI/TIRS	361
27-March-2016	Landsat 8 OLI/TIRS	312
12-April-2016	Landsat 8 OLI/TIRS	265
6-May-2016	Landsat 7 ETM+	232
14-May-2016	Landsat 8 OLI/TIRS	244
22-May-2016	Landsat 7 ETM+	209
15-June-2016	Landsat 8 OLI/TIRS	186
1-July-2016	Landsat 8 OLI/TIRS	248
17-July-2016	Landsat 8 OLI/TIRS	251
25-July-2016	Landsat 7 ETM+	245
10-August-2016	Landsat 7 ETM+	236
11-September-2016	Landsat 7 ETM+	470
19-September-2016	Landsat 8 OLI/TIRS	468
5-October-2016	Landsat 8 OLI/TIRS	334
13-October-2016	Landsat 7 ETM+	334
29-October-2016	Landsat 7 ETM+	335
6-November-2016	Landsat 8 OLI/TIRS	319
14-November-2016	Landsat 7 ETM+	339
8-December-2016	Landsat 8 OLI/TIRS	340
16-December-2016	Landsat 8 OLI/TIRS	266

### 2.5. Hydro-meteorological data and statistical analysis

Monthly and annual runoff and precipitation data (1958–2013), which were recorded at the Langan Hydrological Station in the upper sections of the Keriya River (Fig 1b), were provided by the Hotan Hydrology and Water Resource Survey Bureau of Xinjiang Uygur Autonomous Region, China. Monthly and annual temperature data, precipitation data, and relative humidity data (1961–2019) collected at the middle reach meteorological station, were downloaded from the China Meteorological Data Service Center (http://data.cma.cn/). The middle reach meteorological station is located about 40 km south of the Langan Hydrological Station (Fig 1b). The difference in elevation between the meteorological and hydrological stations is about 500 m. Given the limited elevation difference, the temperature data can be effectively used to represent upper reach areas.

Seven social and economic indicators were also obtained for the analysis of the impacts of anthropogenic disturbances. These data were obtained from the Xinjiang Statistical Yearbook (2001–2020) and included the total population (defined as the number of people in the area), the agricultural population (the total number of people engaged in agricultural activities such as farmers and herdsmen), as well as factors describing agricultural production (sown area of crops, or the total area of land used by the crops harvested during the year), cultivated area (the area of land for planting crops), total drive of agricultural machines (the total rated capacity of all agricultural machinery in the area), and descriptors of the regional economy (regional gross domestic product (GDP) and the value of total agricultural production) (the above definitions are from the Xinjiang Statistical Yearbook). The indices that represent water utilization at the Yutian oasis were also collected, including water usage from springs, wells, reservoirs, and river water (2000–2011) (provided by the Yutian Water Conservancy Bureau of Xinjiang Uygur Autonomous Region, China). Data pertaining to the agricultural water demand in 2009 in the middle reach of the basin were obtained from the Planning Report of Irrigation and Water Conservancy Construction in Yutian County (we only found data for 2009). Data regarding the area irrigated within the Yutian oasis from 2012 to 2017 were obtained from the Xinjiang Statistical Yearbook and the Hotan Statistical Yearbook. The middle reach human water use is defined in this study as the annual quantity of water withdrawn from surface- and groundwater resources.

## 3. Results

### 3.1. Inter-annual and intra-annual variations of river length within the lower reaches of the Keriya River

#### 3.1.1. Inter-annual variations

**Fig 6:**
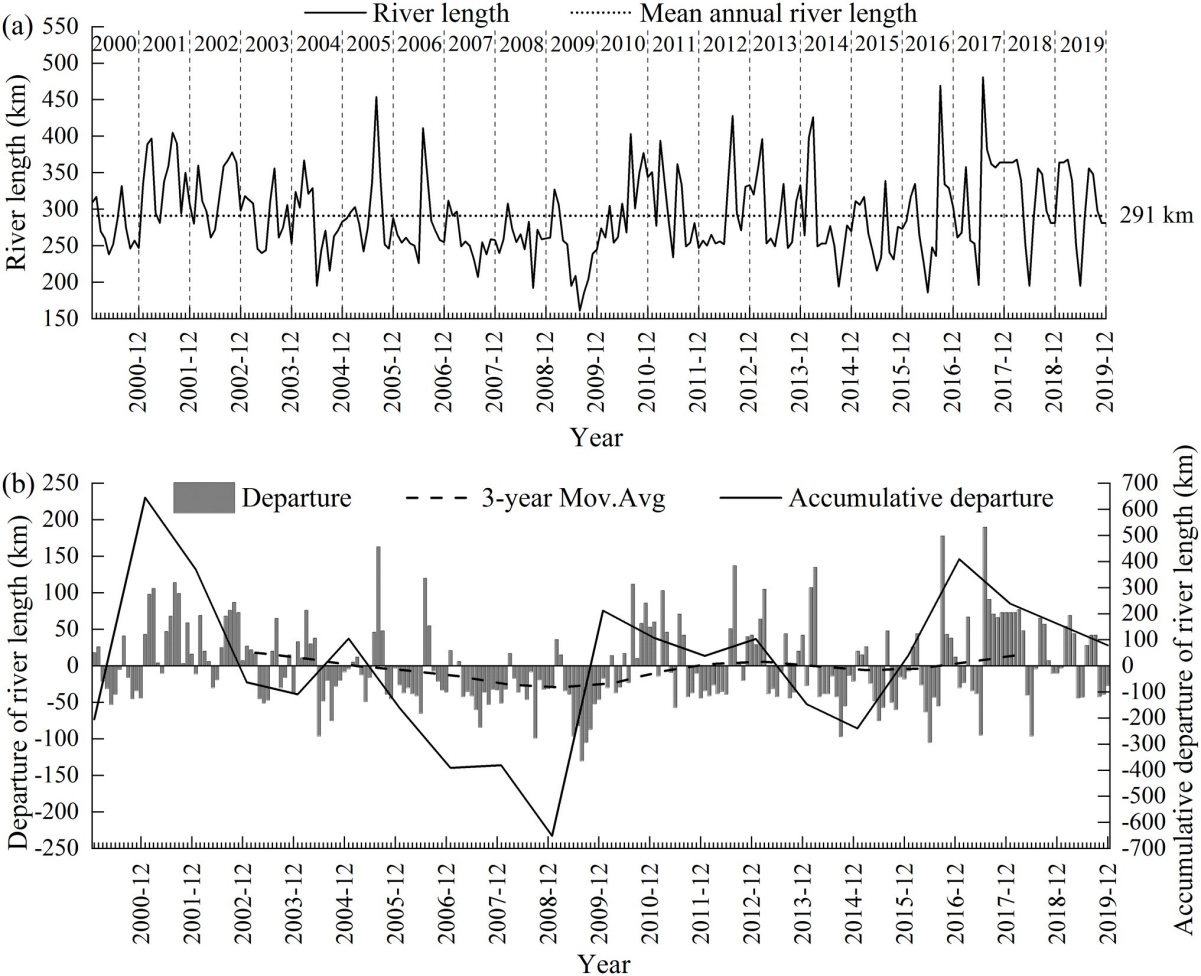
Observed changes in river length measured along the lower reaches of the Keriya River between 2000–2019 (a) and departures, accumulative departure and 3-year moving average of river length from 2000 to 2019 (b). The horizontal axis (X-axis) tick marks show months for each year.

**Fig 7:**
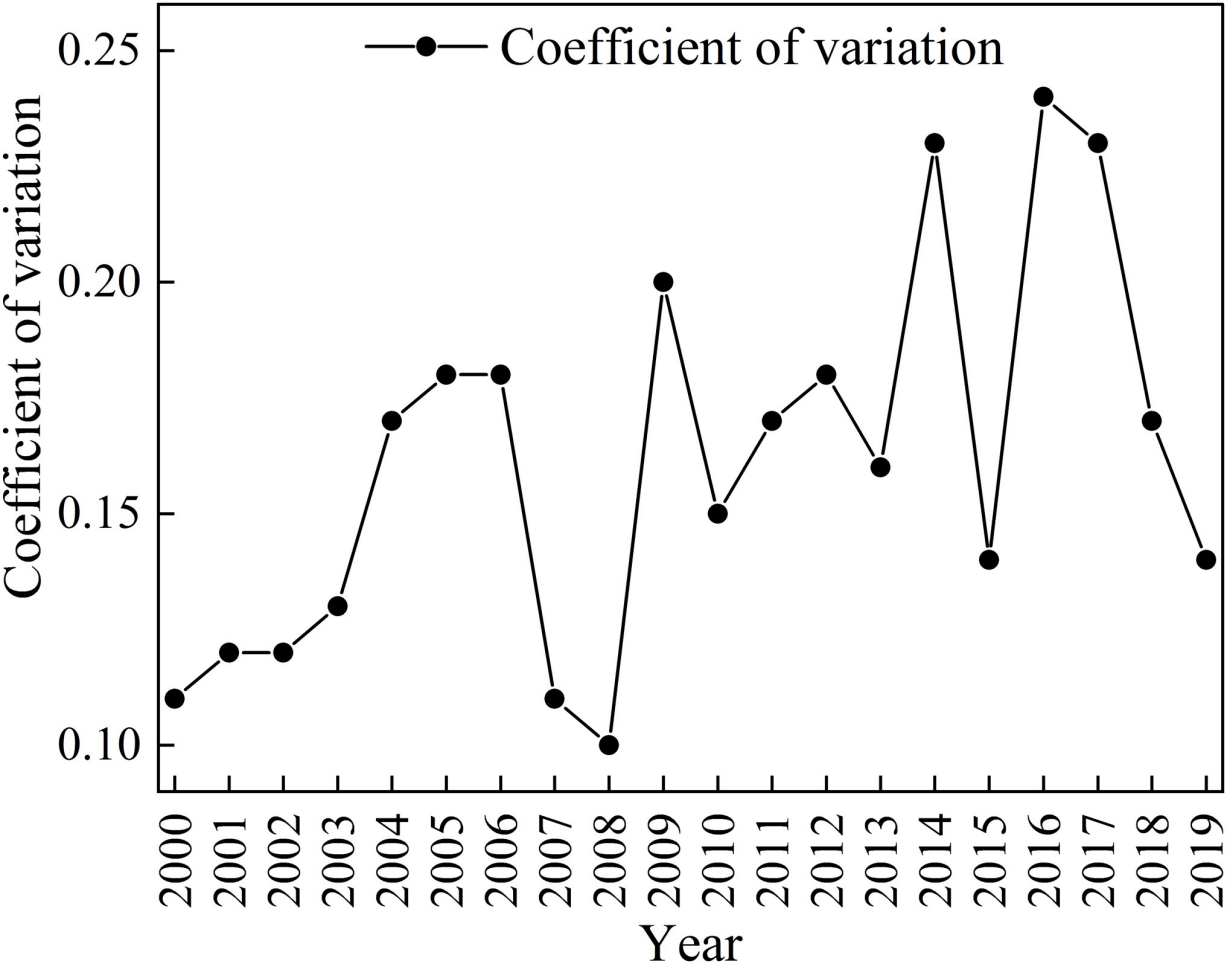
Observed changes in the coefficient of variation of river length from 2000 to 2019.

We obtained 240 monthly river length values from the lower reaches of the Keriya River over a period of 20 years. These values fluctuated around 291 km (the 20-year average between 2000–2019) (Fig 6a). Overall, there is a gradual increase in the amplitude of variations from 2000 to 2019 (Fig 6a). The river length of the Keriya River decreased from 2000 to 2009, before increasing to 2019 (Fig 6a). In 2009, there was a pronounce turning point (at a minimum value) in river length over the past 20 years (Fig 6a); the departure, cumulative departure, and the 3-year moving average all illustrate the nature and timing of the turning point (Fig 6b). The rise and fall around the zero value of departure in river length (Fig 6b) tended to increase toward 2019. However, the overall trend could be subdivided into different sections on either side of 2009. The frequency of years with a negative departure was significantly higher than those with a positive departure before 2009, whereas after 2009, the positive departure markedly increased. This indicates that length of the lower Keriya River was higher than the long-term average annual value (291 km) in most years after 2009. Thus, 2009 appears to represent a turning point in river length over the past 20 years (Fig 6b). The maximum value of departure from the mean occurred in 2001, whereas the minimum value occurred in 2009. The 3-year moving average shows a significant decrease from 2003 to 2009, and a mild increase from 2010 to 2017 (Fig 6b). The coefficient of variation fluctuated markedly between 2000–2019 and varied from 0.1 to 0.24 (Fig 7). The coefficients of variation were relatively high in 2009, 2014, 2016 and 2017, indicating a higher dispersion, which means that river length fluctuations intensified. The value of the coefficients of variation in 2000 and 2008 were relatively small, indicating slightly lower variations in the change in river length during these years.

#### 3.1.2. Intra-annual variation

**Fig 8:**
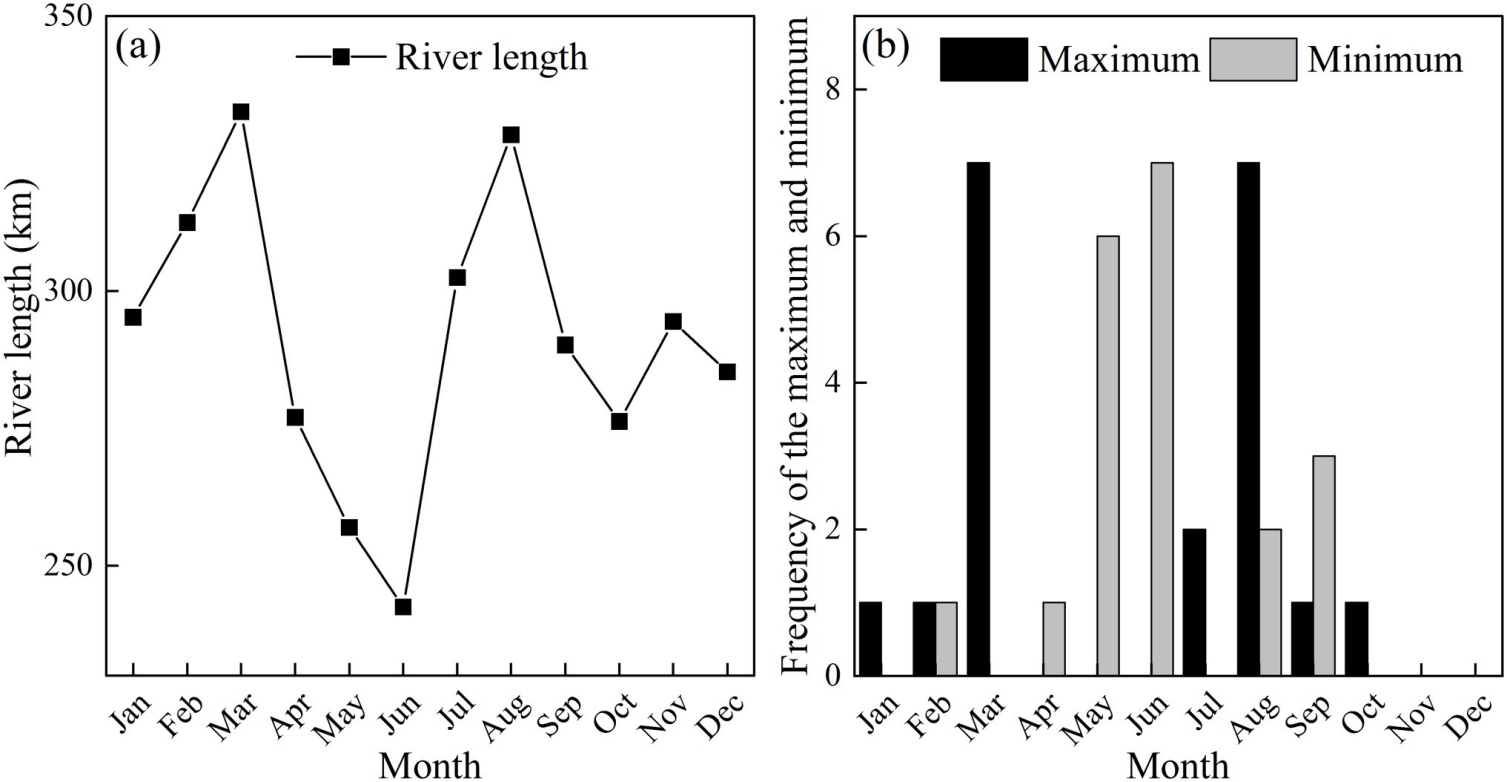
(a) Observed intra-annual changes of the 20-year average river length along the lower reaches of the Keriya River. (b) Occurrence frequency of the minimum and maximum lower reach river length stratified by month (based on data from 2000 to 2019).

Intra-annual variations in the average river length over a 20-year period (from 2000 to 2019) along the lower reaches of the Keriya River also fluctuated (Fig 8a). Three peaks (occurring in March, August and November) and two lows (occurring in June and October) are apparent on the curve (Fig 8a). The annual maximum value of river length between 2000 and 2019 occurred 7 times in March and August, whereas the annual minimum value in river length appeared 6 times in May and 7 times in June. The peak in March was slightly higher than that in August. The lower reach river length weakly changed on the long-term time scale from November to December; no maximum or minimum values occurred during this period (Fig 8b).

### 3.2. Relationship between variations in river length and natural factors

#### 3.2.1. Inter-annual variation analysis

**Fig 9:**
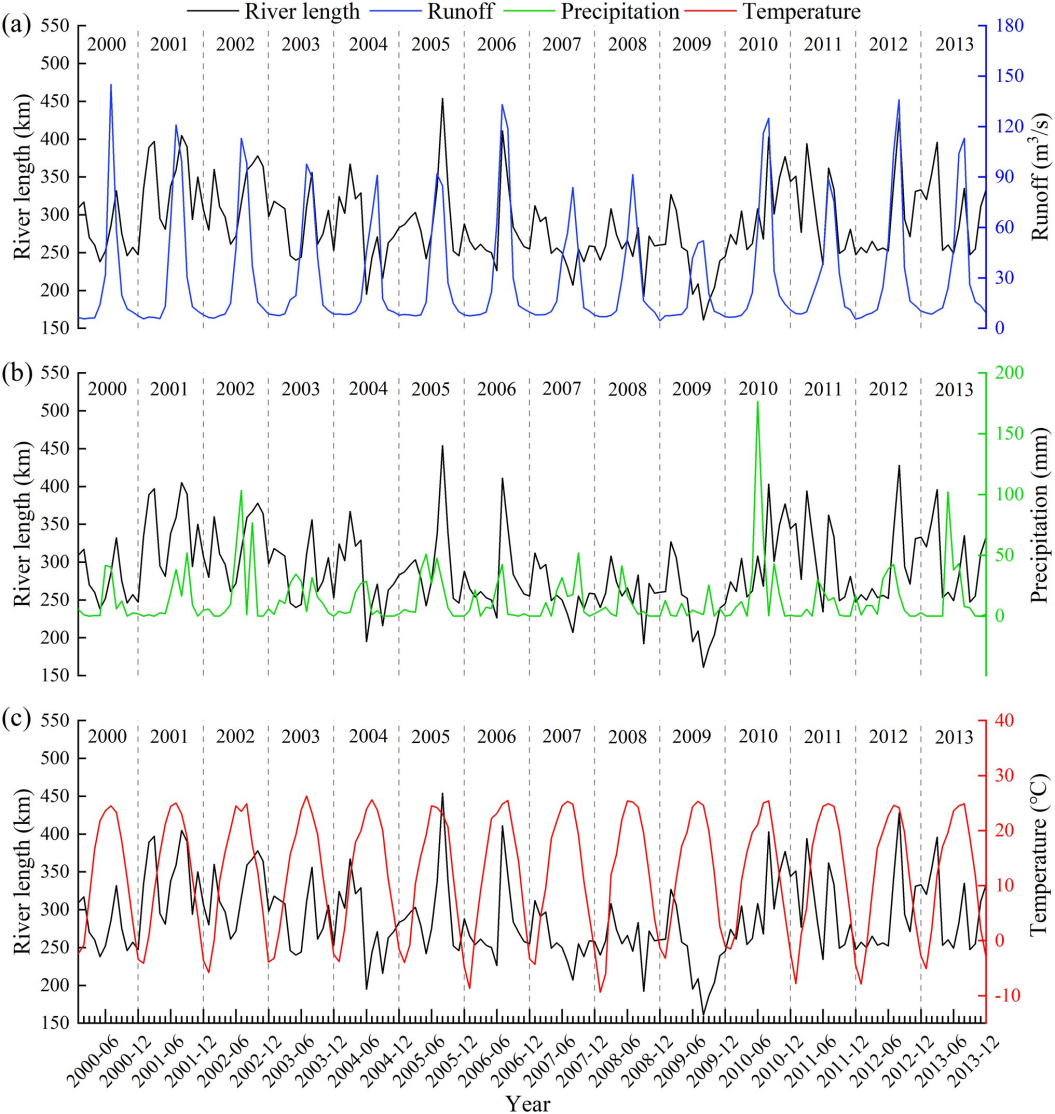
Observed changes between lower reach river length and hydro-meteorological data in the Keriya River Basin. (a), (b) and (c) represent the dynamic changes between lower reach river length and upper reach runoff, precipitation, and temperature from 2000 to 2013, respectively.

River length consistently increased with runoff (*r* = 0.70, *p*<0.01) during the periods of 2000–2006 and 2010–2013 when maximum values of each occurred. The opposite trend occurred between 2007–2009 (Fig 9a); river length reached a peak while upper reach runoff was relatively low. River length and precipitation were positively correlated (*r* = 0.55, *p*<0.05), especially during the periods of 2001–2003, 2005–2006 and 2010. However, in 2000, 2004, 2007–2009 and 2011–2013, precipitation and river length were negatively correlated, as the peak values of river length corresponded to lows in precipitation (Fig 9b). River length was negatively correlated to air temperature (*r* = −0.44), especially between 2001–2004 and 2007–2009, however, inconsistencies were identified in 2000, 2005–2006 and 2010–2013 (Fig 9c).

#### 3.2.2. Intra-annual variation analysis

**Fig 10:**
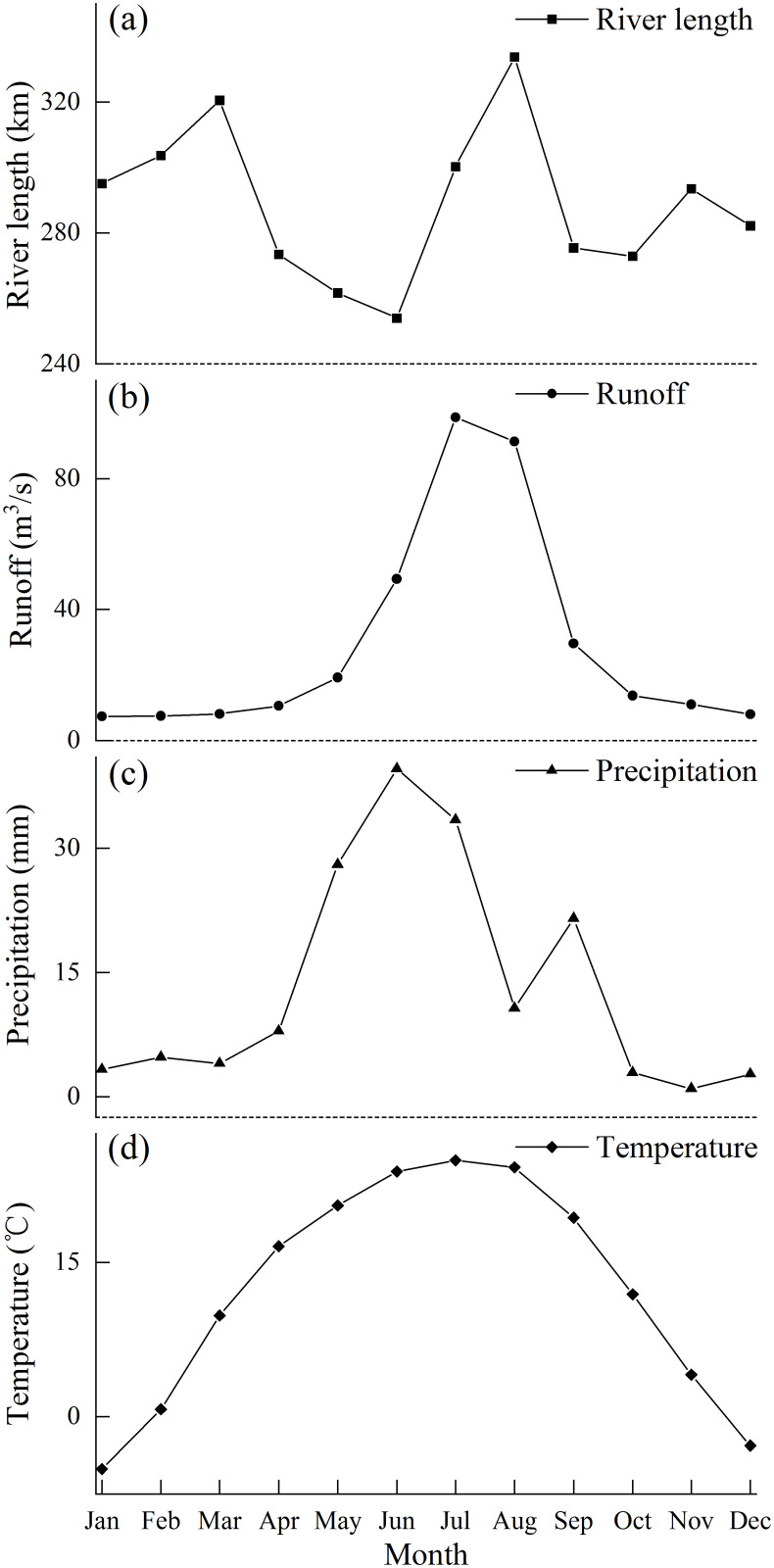
Observed intra-annual changes between lower reach river length and natural upper reach factors in the Keriya River Basin. (a), (b), (c) and (d) represent the seasonal changes in lower reach river length, upper reach runoff, precipitation and temperature, respectively.

Although the average (2000–2013) monthly lower reach river length, upper reach runoff, precipitation and temperature were calculated for a period of only 14 years, the intra-annual variations are clear. All four of these parameters fluctuated seasonally (Fig 10). More specifically, precipitation occurs primarily from May to September. Two peaks, one in June and one in September, were identified during these months, along with a minimum value in August (Fig 10c). The variations in upper reach runoff are characterized by a single peak in July (Fig 10b). Runoff was concentrated during the summer (from June to August). Temperature began to rise in March, and the highest peak formed in July (Fig 10d). River length was elevated from July to August (Fig 10a) and lagged approximately two months behind the peak in precipitation; runoff was also elevated during this time. The runoff peaks were consistent with temperature (both occurring in July) and lagged approximately one month behind the peak in precipitation.

### 3.3. Relationships between river length and anthropogenic disturbances

#### 3.3.1. Inter-annual variation analysis

**Fig 11:**
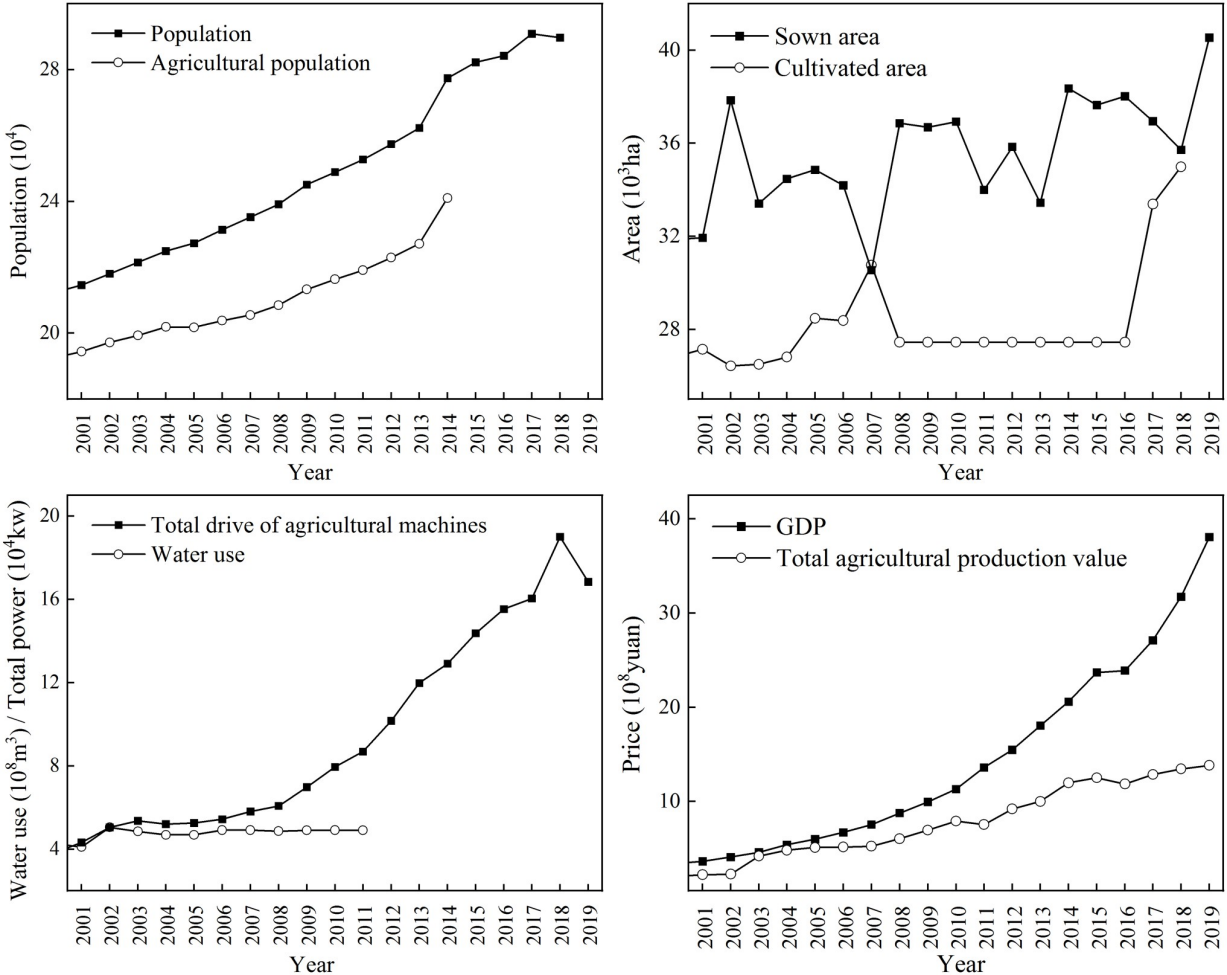
Social and economic changes in Yutian County, located within the middle reaches of the Keriya River from 2000 to 2019, including population (a), agricultural population (a), sown area (b), cultivated area (b), total drive of agricultural machines (c), water use (c), GDP (d), and total agricultural production (d).

The indices we used to represent anthropogenic disturbances in the Yutian oasis included total population, agricultural population, cultivated area, sown area, total drive of agricultural machines, amount of water consumption, GDP and total agricultural production in the middle reaches of the Keriya River. The values of these parameters increased from 2000 to 2019, and most increased linearly (Fig 11), while the river length fluctuated significantly. Therefore, these indicators were inconsistent with the inter-annual variations in lower reach river length (Fig 6a); the correlation coefficients between them and lower reach river length were insignificant at the 95% confidence level (*p*<0.05) (Table 4).

**Table 4:** Correlation results between river length and anthropogenic disturbances in the Keriya River Basin.

Parameter	Period	Correlation coefficient (*r*)
Population	2000–2018	0.06
Agricultural population	2000–2014	−0.16
Sown area	2000–2019	0.2
Cultivated area	2000–2019	0.03
Total drive of agricultural machines	2000–2019	0.2
Water use	2000–2011	−0.35
GDP	2000–2019	0.16
Total agricultural production value	2000–2019	0.05

#### 3.3.2. Intra-annual variation analysis

**Fig 12:**
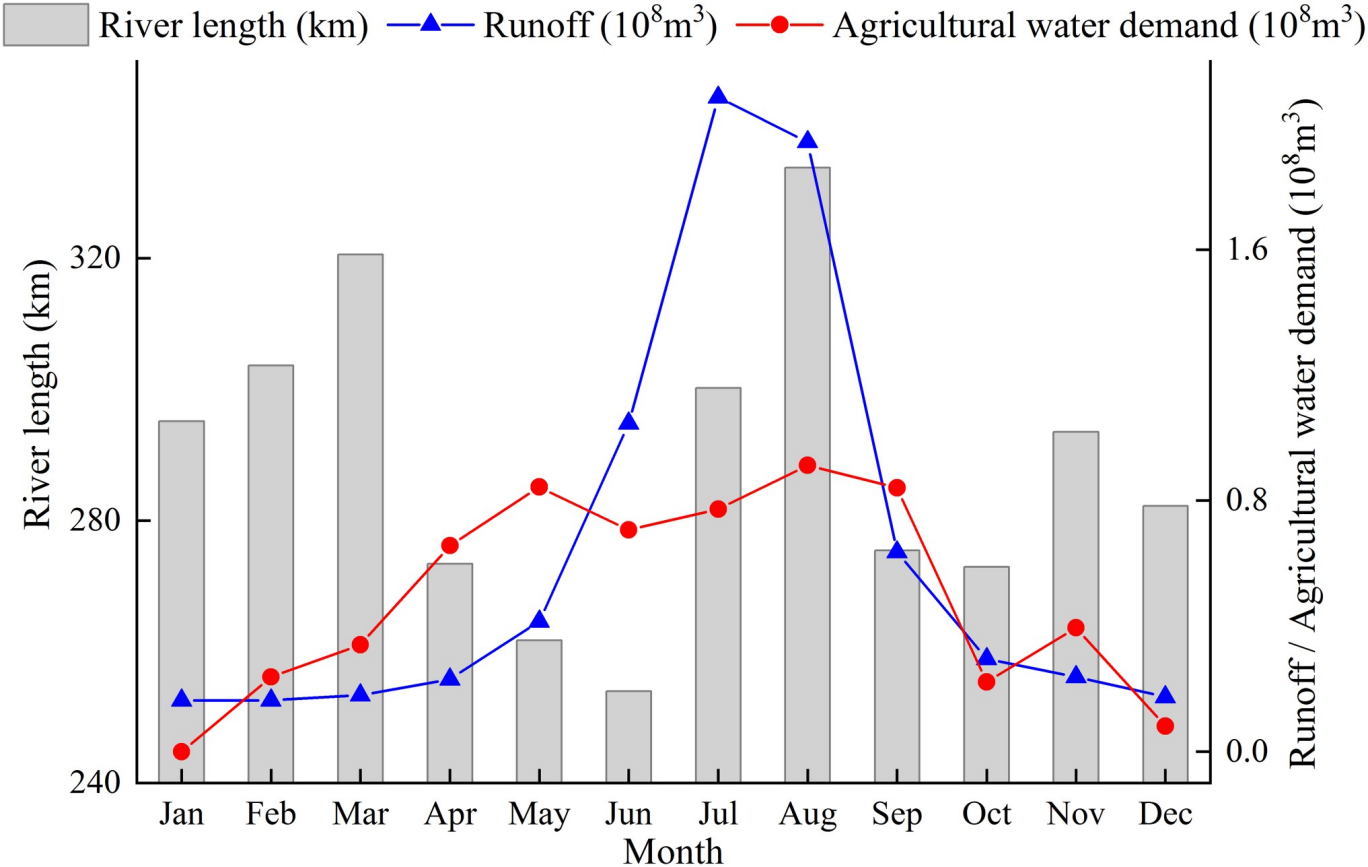
Observed intra-annual changes in lower reach river length (20-year average), upper reach runoff (14-year average) and middle reach agricultural water demand (in 2009) of the Keriya River.

Intra-annual variations in the average lower reach river length during the 20-year study period (from 2000 to 2019) and the middle reach agricultural water demand in 2009 both fluctuated seasonally. Three peaks and two minimum values were both identified from the two curves (Fig 12). Peak values in river length occurred in March, August and November, whereas peak values in agricultural water demand occurred in May, August and November. The low values in river length and agricultural water demand both occurred in June and October. Agricultural water demand increased significantly from April to May, while the river length decreased sharply, before both reached a minimum value in June. They then increased from July to August, before declining from September to October. A small peak in both formed in November.

## 4. Discussion

### 4.1. Factors influencing lower reach river length

The length changes in IRES in dryland regions mainly occur along their lower reaches [6], especially in extremely arid desert regions; these lower reach variations in river length should be directly related to the recharge in surface runoff (Fig 1c). Tang et al. [57] suggested that lower reach changes in flow are greatly affected by runoff in the headwaters. In the current study, a significant correlation between a change in length of the lower Keriya River and runoff in the upper reach mountains (*r* = 0.70, *p*<0.01), combined with the consistency between the inter-annual and intra-annual variations in river length and upper reach runoff in summer (Figs 9a and 12), supports the argument by Tang et al. [57]. In terms of runoff in mountainous headwater regions, the impact of humans is limited because of its low population density. Thus, runoff processes in the mountains are mostly controlled by natural factors such as climate change; the most important climatic factors are precipitation and temperature [62] (Fig 9). The intra-annual and inter-annual variations in runoff generally lag behind precipitation (Figs 9 and 10). This suggests precipitation should play an important recharge role in controlling runoff in the mountainous areas, which is consistent with a positive correlation (*r* = 0.66, *p*<0.05) in the inter-annual changes between them. The positive correlation between upper reach runoff and precipitation is broadly consistent with the results presented for the Tarim River Basin [76–78]. Regarding the impact of temperature on runoff, Chen [63] and Zhou et al. [62] believed that the rise in air temperature causes a significant increase in glacial and snowmelt runoff in mountainous areas; Ling et al. [78, 79] found that runoff was significantly positively correlated with temperature. Our data showed that the correlation between them was negatively correlated (*r* = −0.52), which may be related to an insufficient time series in this study. In conclusion, upper reach natural factors, including runoff, precipitation, and temperature, should affect lower reach variations in river length.

**Fig 13:**
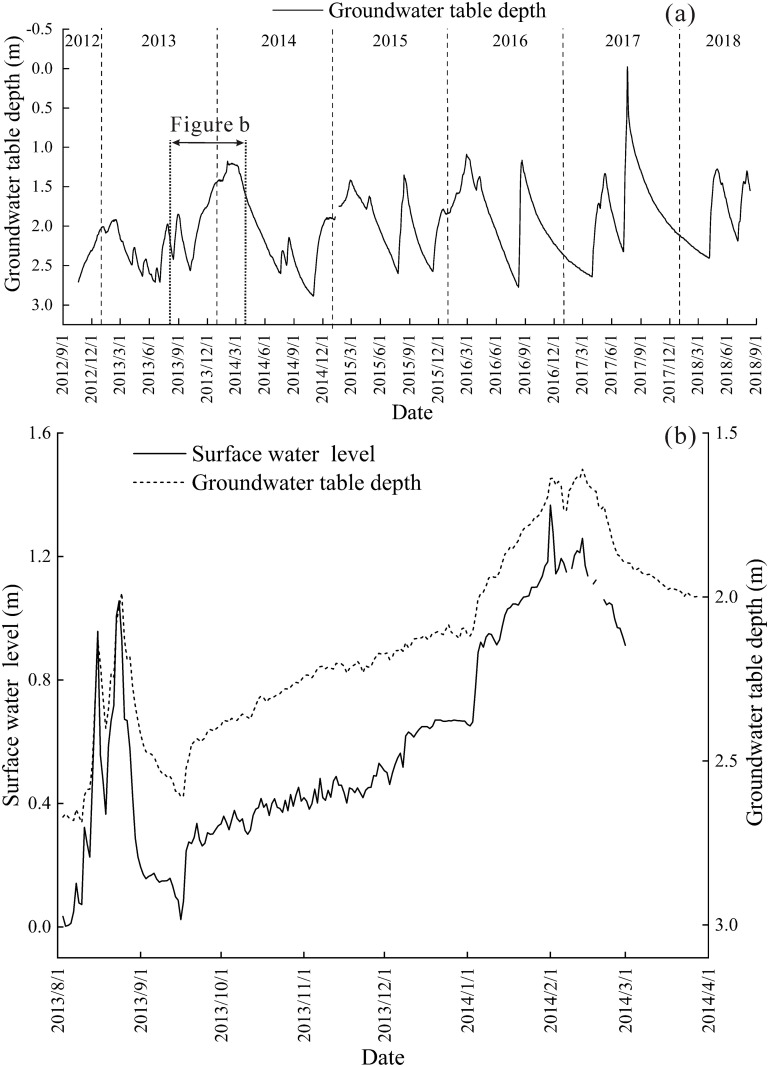
(a) Dynamic variation in the depth to the groundwater table in the monitoring well in Daliyaboyi oasis (based on data from 2012 to 2018); (b) Dynamic variation of the surface water level and groundwater table depth in the monitoring well in the upstream section of the lower reach (based on data from 2013 to 2014) [80, 81].

The artificial Yutian agricultural oasis in the middle reach area is the dominant zone of water consumption along the Keriya River Basin [72]. Agriculture in the Yutian oasis primarily consumes surface runoff that flows to lower reach areas, which affects variations in river length along the lower reaches. The total amount of irrigation water consumed in Yutian oasis reached 4.11–5.05×10^8^ m^3^ yr –^1^ between 2000–2011 (Fig 11). The removal of water for irrigation decreased about 63% of the water discharged to the lower reach area (Table 5). This indicates that variations in lower reach river length are mainly affected by natural headwater factors and middle reach anthropogenic disturbances. In addition, according to dynamic changes in water table depth, groundwater (discharged from the Yutian oasis in the middle reaches) replenished lower reach surface runoff [80, 81] (Fig 13) and the influx of melt-water from ice along lower reach river channels, which both affected changes in river length.

**Table 5:** Calculation and description of upper reach runoff and water use in the Yutian oasis.

Parameter	Formula	Description
Percentage of total runoff consumed by the Yutian oasis	$A=\frac{W1}{R}\times 100\%$	*A* is the percentage of total runoff consumed by the Yutian oasis; *W*1 is mean annual irrigation water consumed in the Yutian oasis, or 4.76×10^8^ m^3^ (2000–2011); *R* is the mean annual runoff (7.57×10^8^ m^3^) (1958–2013); *B* is the summer runoff in the upper reaches; *W*2 is the proportion total annual runoff by summer flows (66.4%); *C* is the summer water consumption in the Yutian oasis; *W*3 is the proportion of total annual water consumption during the summer in the Yutian oasis (53%).
Summer runoff in the upper reaches	B=W2×R	
Summer water consumption in the Yutian oasis	C=W3×R	

Note: calculations are based on Chen [63].

### 4.2. Inter-annual variation analysis of river length

The minimum in length of the lower Keriya River occurred in 2009 (Fig 6a), which coincides with upper reach runoff along the Keriya River (Fig 9a). Upper reach runoff of other rivers in the Tarim Basin includes the Aksu River, the Yarkand River, the Hotan River, and the Qarqan River [46, 55, 56, 82–90], all of which exhibited similar variations in runoff and exhibited a minimum in 2009. It can be concluded that the rivers within the Tarim Basin generally experienced low runoff in 2009. We also compared runoff within the Keriya River Basin with the main rivers and lakes of Central Asia (including the Amu Darya, Syr Darya, Aral Sea, and Issyk-Kul Lake), and found the lowest value in 2009 in the Aral Sea (water surface area) [91–93]. The Amu Darya, Syr Darya (upstream runoff) and Issyk-Kul Lake (water surface area) exhibited a minimum in 2008 [94–97]. As mentioned above in section 4.1, large-scale agricultural irrigation in the middle reaches usually leads to lower reach decreases in runoff, which may have led to the low in 2009. However, the use of irrigation water along the middle reaches of the Keriya River (Fig 11) and other rivers in Tarim Basin [55] have increased slightly since 2000, in spite of an absence of an abrupt large increase in water abstractions. The agricultural economic indicators in the middle reach oasis are also inconsistent with the inter-annual variations in lower reach river length (Figs 6a and 11); the correlations between them and lower reach river length are surprisingly low (−0.35<*r*<0.03) and are not statistically significant (Table 4). These relations imply that middle reach anthropogenic disturbances have a limited impact on the variations in lower reach river length.

### 4.3. Intra-annual variation analysis on river length

Seasonal variations (Fig 10a) imply that there are different impacts between upper reach natural factors (Fig 10b–10d) and middle reach anthropogenic disturbances (Fig 12) on the length of the lower Keriya River. The upper reach runoff dropped to a low value in March (Fig 10b); in contrast, the lower reach river length increased to a peak value (Fig 10a). Two possible reasons for this inconsistency include: (1) the two highest peaks in groundwater levels occurred in February-March of the year during the monitoring period (from 2012 to 2018) in the Daliyaboyi oasis (Fig 13a) and during the monitoring period (from 2013 to 2014) in the upstream section of the lower reach (Fig 13b) respectively [80, 81], which suggests that groundwater may have recharged downstream runoff; and (2) air temperature begins to climb in March (Fig 10d), and therefore ice along the river will gradually melt begins in the lower reaches and continue continues into the middle reaches in accordance with elevation (Fig 1c). The approximately 200–400 m decrease in elevation from the south (middle reach Yutian oasis) to the north (lower reach Daliyaboyi oasis) (Fig 1c) should facilitate the influx of meltwater and groundwater to the channel. In summer, the marked rise in air temperature (Fig 10d) causes a significant increase in glacial and snowmelt runoff in the mountainous areas [63] (Fig 10b). Rainfall in mountainous areas also significantly contributes to upper reach runoff [63]. These two natural factors may cause the upper reach runoff to reach its maximum value of approximately 5.02×10^8^ m^3^ [63] (Table 5). Intra-annual variation in middle reach water consumption also reaches its highest peak (Fig 12) during the summer, which was approximately 4.01×10^8^ m^3^ (the peaks mainly occurred in August) [63] (Table 5). Agricultural water consumption in areas along the middle reach reduced the water discharge to lower reach areas by about 80%. The peak value in river length occurred in summer because (1) the peak values in upper reach runoff and middle reach water consumption both occurred in summer, and the former is higher than the latter by about 20% in summer; and (2) the inter-annual variation (12-year) in middle reach water use changed steadily (Fig 11). Although there are missing data on middle reach water consumption from 2012 to 2019, data on population, cultivated area, and effective irrigated area after 2011 show that the inter-annual variations in middle reach water consumption in recent years were not significant. Moreover, these trends show that seasonal variations in river length reflect the frequency and amplitude of fluctuations in upper reach runoff (Fig 12). Human water consumption affects the fluctuation magnitude of river length to a certain extent, which is well illustrated by the lowest value in river length occurring in June. The minimum value in June (Fig 10a) might be related to both the decrease in upper reach runoff (Fig 10b) and large-scale irrigation in the middle reach areas (Fig 12). The data show a small peak in river length in November of each year (Fig 10a). This minor increase in river length may be caused by decreasing truncation of autumn floods in the middle reach areas (Fig 12), which increases the amount of river water that flows to lower reach areas. Although there is only one year of agricultural water demand data, the inter-annual variations in water consumption are minor (Fig 11); thus, the data in this study is representative.

Natural factors in the upper reaches mainly control trends in the overall inter-annual and intra-annual variations in the magnitude of river length of the Keriya River (Figs 9, 11 and 12), though anthropogenic disturbances in the middle reaches contribute to the fluctuations. Nevertheless, with an increase in the development of water conservation technology, the anthropogenic impacts on lower reach runoff processes will increase [98], whereas the impact of natural factors will gradually weaken. These changes are well-illustrated by variations in length of the lower Keriya River after 2018, when the Jiyin Reservoir within the upper reach (Fig 1b) was put into use. The removal of the flood peak discharge during the summer within the lower reaches of the Keriya River from 2018 to 2019 (Fig 6a) is related to the regulation of river flow by the Jiyin Reservoir.

## 5. Conclusions

River length can be used as a novel indicator to reveal the dynamic variations in lower reach surface runoff in IRES in dryland areas. We digitized and measured the distance (river length) between the center of Yutian County and the river’s end point on 272 remote sensing images and then constructed monthly lower reach inter-annual and intra-annual variations in length of the lower Keriya River over a 20-year period (2000–2019). The results showed that: (1) upper reach runoff, the quantity of water consumed by humans along the middle reaches, and groundwater and meltwater influx along lower reach river channels all contribute to the intra-annual variations in length (20-year average) of the lower Keriya River. The minimum value in river length occurred in June, because of the increase in middle reach agricultural irrigation and a low in upper reach runoff. The peak in river length occurred in August because of increased upper reach runoff, despite the maximum values of agricultural water demand also occurring at the same time within the year. The former (upper reach runoff) is about 20% higher than the latter in summer. In March, the peak value in river length may be due to elevated lower reach groundwater levels and the influx of meltwater from ice along river channels; (2) the overall trends in inter-annual variations in river length, including the frequency and amplitude of its fluctuations, are closely correlated with upper reach changes in runoff as well as middle reach water consumption, which increased slightly during the study period. The inter-annual variations in the frequency and amplitude of fluctuations in river length are mainly controlled by upper reach runoff. In addition, the lowest value in river length occurred in 2009, consistent with the low value in upper reach runoff of the Keriya River and other rivers in the Tarim Basin. Collectively, natural factors control the inter-annual and intra-annual variations in length of the lower Keriya River. This research differs from previous studies that focused exclusively on the impacts of anthropogenic disturbances on surface runoff within the Tarim River Basin.

## Supporting information

S1 Table Information on the Landsat imagery used. (DOCX)
