# Supplementary material for: Influence of natural and anthropogenic controls on runoff in the Keriya River, central Tarim Basin, China
Source: PLoS One. 2022 May 27;17(5):e0269132. doi: 10.1371/journal.pone.0269132 (PMC9140297; doi:10.1371/journal.pone.0269132)
Supplement: S1 Table — (DOCX) [file pone.0269132.s001.docx]

**S1 Table. Information on the Landsat imagery used.** TM: Thematic Mapper, ETM+: Enhanced Thematic Mapper Plus, OLI_TIRS: Operational Land Imager (OLI) and Thermal Infrared Sensor (TIRS).

| Acquisition date | Sensor | Acquisition date | Sensor |
| --- | --- | --- | --- |
| 20-February-2000 | Landsat 7 ETM+ | 23-March-2000 | Landsat 7 ETM+ |
| 10-May-2000 | Landsat 7 ETM+ | 27-June-2000 | Landsat 7 ETM+ |
| 30-August-2000 | Landsat 7 ETM+ | 17-October-2000 | Landsat 7 ETM+ |
| 5-January-2001 | Landsat 7 ETM+ | 22-February-2001 | Landsat 7 ETM+ |
| 10-March-2001 | Landsat 7 ETM+ | 11-April-2001 | Landsat 7 ETM+ |
| 29-May-2001 | Landsat 7 ETM+ | 30-June-2001 | Landsat 7 ETM+ |
| 2-September-2001 | Landsat 7 ETM+ | 5-November-2001 | Landsat 7 ETM+ |
| 8-January-2002 | Landsat 7 ETM+ | 25-February-2002 | Landsat 7 ETM+ |
| 29-March-2002 | Landsat 7 ETM+ | 16-May-2002 | Landsat 7 ETM+ |
| 3-July-2002 | Landsat 7 ETM+ | 20-August-2002 | Landsat 7 ETM+ |
| 7-October-2002 | Landsat 7 ETM+ | 8-November-2002 | Landsat 7 ETM+ |
| 26-December-2002 | Landsat 7 ETM+ | 11-January-2003 | Landsat 7 ETM+ |
| 12-February-2003 | Landsat 7 ETM+ | 12-July-2003 | Landsat 7 ETM+ |
| 26-October-2003 | Landsat 7 ETM+ | 27-November-2003 | Landsat 7 ETM+ |
| 14-January-2004 | Landsat 7 ETM+ | 2-March-2004 | Landsat 7 ETM+ |
| 3-April-2004 | Landsat 7 ETM+ | 5-May-2004 | Landsat 7 ETM+ |
| 6-June-2004 | Landsat 7 ETM+ | 8-July-2004 | Landsat 7 ETM+ |
| 2-August-2004 | Landsat 7 ETM+ | 26-September-2004 | Landsat 7 ETM+ |
| 13-November-2004 | Landsat 7 ETM+ | 15-December-2004 | Landsat 7 ETM+ |
| 16-January-2005 | Landsat 7 ETM+ | 1-February-2005 | Landsat 7 ETM+ |
| 5-March-2005 | Landsat 7 ETM+ | 22-April-2005 | Landsat 7 ETM+ |
| 8-May-2005 | Landsat 7 ETM+ | 25-June-2005 | Landsat 7 ETM+ |
| 27-July-2005 | Landsat 7 ETM+ | 12-August-2005 | Landsat 7 ETM+ |
| 13-September-2005 | Landsat 7 ETM+ | 15-October-2005 | Landsat 7 ETM+ |
| 18-December-2005 | Landsat 7 ETM+ | 24-March-2006 | Landsat 7 ETM+ |
| 25-April-2006 | Landsat 7 ETM+ | 20-June-2006 | Landsat 4-5 TM |
| 22-July-2006 | Landsat 4-5 TM | 31-August-2006 | Landsat 7 ETM |
| 24-September-2006 | Landsat 4-5 TM | 10-October-2006 | Landsat 4-5 TM |
| 3-November-2006 | Landsat 7 ETM+ | 21-December-2006 | Landsat 7 ETM+ |
| 6-January-2007 | Landsat 7 ETM+ | 23-February-2007 | Landsat 7 ETM+ |
| 27-March-2007 | Landsat 7 ETM+ | 28-April-2007 | Landsat 7 ETM+ |
| 6-May-2007 | Landsat 4-5 TM | 22-May-2007 | Landsat 4-5 TM |
| 23-June-2007 | Landsat 4-5 TM | 9-July-2007 | Landsat 4-5 TM |
| 10-August-2007 | Landsat 4-5 TM | 11-September-2007 | Landsat 4-5 TM |
| 27-September-2007 | Landsat 4-5 TM | 6-November-2007 | Landsat 7 ETM+ |
| 24-December-2007 | Landsat 7 ETM+ | 26-February-2008 | Landsat 7 ETM+ |
| 13-March-2008 | Landsat 7 ETM+ | 22-April-2008 | Landsat 4-5 TM |
| 8-May-2008 | Landsat 4-5 TM | 1-June-2008 | Landsat 4-5 TM |
| 25-June-2008 | Landsat 7 ETM+ | 3-July-2008 | Landsat 7 ETM+ |
| 20-August-2008 | Landsat 7 ETM+ | 5-September-2008 | Landsat 7 ETM+ |
| 23-October-2008 | Landsat 7 ETM+ | 31-October-2008 | Landsat 4-5 TM |
| 24-November-2008 | Landsat 7 ETM+ | 26-December-2008 | Landsat 4-5 TM |
| 11-January-2009 | Landsat 4-5 TM | 4-February-2009 | Landsat 4-5 TM |
| 8-March-2009 | Landsat 4-5 TM | 25-April-2009 | Landsat 4-5 TM |
| 27-May-2009 | Landsat 4-5 TM | 28-June-2009 | Landsat 4-5 TM |
| 6-July-2009 | Landsat 7 ETM+ | 15-August-2009 | Landsat 4-5 TM |
| 16-September-2009 | Landsat 4-5 TM | 2-October-2009 | Landsat 4-5 TM |
| 18-October-2009 | Landsat 4-5 TM | 26-October-2009 | Landsat 7 ETM+ |
| 3-November-2009 | Landsat 4-5 TM | 19-November-2009 | Landsat 7 ETM+ |
| 29-December-2009 | Landsat 7 ETM+ | 22-January-2010 | Landsat 4-5 TM |
| 7-February-2010 | Landsat 4-5 TM | 11-March-2010 | Landsat 4-5 TM |
| 28-April-2010 | Landsat 4-5 TM | 29-May-2010 | Landsat 7 ETM+ |
| 23-June-2010 | Landsat 7 ETM+ | 17-July-2010 | Landsat 4-5 TM |
| 10-August-2010 | Landsat 7 ETM+ | 26-August-2010 | Landsat 7 ETM+ |
| 11-September-2010 | Landsat 7 ETM+ | 13-October-2010 | Landsat 7 ETM+ |
| 21-October-2010 | Landsat 4-5 TM | 29-October-2010 | Landsat 7 ETM+ |
| 22-November-2010 | Landsat 4-5 TM | 8-December-2010 | Landsat 4-5 TM |
| 24-December-2010 | Landsat 4-5 TM | 9-January-2011 | Landsat 4-5 TM |
| 14-March-2011 | Landsat 4-5 TM | 15-April-2011 | Landsat 4-5 TM |
| 17-May-2011 | Landsat 4-5 TM | 18-June-2011 | Landsat 4-5 TM |
| 28-July-2011 | Landsat 7 ETM+ | 5-August-2011 | Landsat 4-5 TM |
| 21-August-2011 | Landsat 4-5 TM | 22-September-2011 | Landsat 4-5 TM |
| 16-October-2011 | Landsat 7 ETM+ | 9-November-2011 | Landsat 4-5 TM |
| 4-January-2012 | Landsat 7 ETM+ | 25-April-2012 | Landsat 7 ETM+ |
| 11-May-2012 | Landsat 7 ETM+ | 12-June-2012 | Landsat 7 ETM+ |
| 30-July-2012 | Landsat 7 ETM+ | 31-August-2012 | Landsat 7 ETM+ |
| 16-September-2012 | Landsat 7 ETM+ | 2-October-2012 | Landsat 7 ETM+ |
| 19-November-2012 | Landsat 7 ETM+ | 21-December-2012 | Landsat 7 ETM+ |
| 6-January-2013 | Landsat 7 ETM+ | 7-February-2013 | Landsat 7 ETM+ |
| 11-March-2013 | Landsat 7 ETM+ | 12-April-2013 | Landsat 7 ETM+ |
| 20-April-2013 | Landsat 8 OLI/TIRS | 22-May-2013 | Landsat 8 OLI/TIRS |
| 7-June-2013 | Landsat 8 OLI/TIRS | 25-July-2013 | Landsat 8 OLI/TIRS |
| 26-August-2013 | Landsat 8 OLI/TIRS | 11-September-2013 | Landsat 8 OLI/TIRS |
| 5-October-2013 | Landsat 7 ETM+ | 21-October-2013 | Landsat 7 ETM+ |
| 30-November-2013 | Landsat 8 OLI/TIRS | 16-December-2013 | Landsat 8 OLI/TIRS |
| 25-January-2014 | Landsat 7 ETM+ | 2-February-2014 | Landsat 8 OLI/TIRS |
| 10-February-2014 | Landsat 7 ETM+ | 6-March-2014 | Landsat 8 OLI/TIRS |
| 23-April-2014 | Landsat 8 OLI/TIRS | 25-May-2014 | Landsat 8 OLI/TIRS |
| 26-June-2014 | Landsat 8 OLI/TIRS | 12-July-2014 | Landsat 8 OLI/TIRS |
| 28-July-2014 | Landsat 8 OLI/TIRS | 5- August-2014 | Landsat 7 ETM+ |
| 30-September-2014 | Landsat 8 OLI/TIRS | 16-October-2014 | Landsat 8 OLI/TIRS |
| 1-November-2014 | Landsat 8 OLI/TIRS | 17-November-2014 | Landsat 8 OLI/TIRS |
| 3-December-2014 | Landsat 8 OLI/TIRS | 20-January-2015 | Landsat 8 OLI/TIRS OLI/TIRS |
| 5-February-2015 | Landsat 8 OLI/TIRS | 1-March-2015 | Landsat 7 ETM+ |
| 26-April-2015 | Landsat 8 OLI/TIRS | 28-May-2015 | Landsat 8 OLI/TIRS |
| 13-June-2015 | Landsat 8 OLI/TIRS | 29-June-2015 | Landsat 8 OLI/TIRS |
| 15-July-2015 | Landsat 8 OLI/TIRS | 16-August-2015 | Landsat 8 OLI/TIRS |
| 17-September-2015 | Landsat 8 OLI/TIRS | 3-October-2015 | Landsat 8 OLI/TIRS |
| 20-November-2015 | Landsat 8 OLI/TIRS | 6-December-2015 | Landsat 8 OLI/TIRS |
| 7-January-2016 | Landsat 8 OLI/TIRS | 31-January-2016 | Landsat 7 ETM+ |
| 8-February-2016 | Landsat 8 OLI/TIRS | 16-February-2016 | Landsat 7 ETM+ |
| 24-February-2016 | Landsat 8 OLI/TIRS | 3-March-2016 | Landsat 7 ETM+ |
| 11-March-2016 | Landsat 8 OLI/TIRS | 27-March-2016 | Landsat 8 OLI/TIRS |
| 12-April-2016 | Landsat 8 OLI/TIRS | 6-May-2016 | Landsat 7 ETM+ |
| 14-May-2016 | Landsat 8 OLI/TIRS | 22-May-2016 | Landsat 7 ETM+ |
| 15-June-2016 | Landsat 8 OLI/TIRS | 1-July-2016 | Landsat 8 OLI/TIRS |
| 17-July-2016 | Landsat 8 OLI/TIRS | 25-July-2016 | Landsat 7 ETM+ |
| 10-August-2016 | Landsat 7 ETM+ | 11-September-2016 | Landsat 7 ETM+ |
| 19-September-2016 | Landsat 8 OLI/TIRS | 5-October-2016 | Landsat 8 OLI/TIRS |
| 13-October-2016 | Landsat 7 ETM+ | 29-October-2016 | Landsat 7 ETM+ |
| 6-November-2016 | Landsat 8 OLI/TIRS | 14-November-2016 | Landsat 7 ETM+ |
| 8-December-2016 | Landsat 8 OLI/TIRS | 16-December-2016 | Landsat 8 OLI/TIRS |
| 1- January-2017 | Landsat 7 ETM+ | 10-February-2017 | Landsat 8 OLI/TIRS |
| 6-March-2017 | Landsat 7 ETM+ | 15-April-2017 | Landsat 8 OLI/TIRS |
| 17-May-2017 | Landsat 8 OLI/TIRS | 18-June-2017 | Landsat 8 OLI/TIRS |
| 28-July-2017 | Landsat 7 ETM+ | 13-August-2017 | Landsat 7 ETM+ |
| 22-September-2017 | Landsat 8 OLI/TIRS | 24-October-2017 | Landsat 8 OLI/TIRS |
| 25-November-2017 | Landsat 8 OLI/TIRS | 3-December-2017 | Landsat 7 ETM+ |
| 12- January-2018 | Landsat 8 OLI/TIRS | 5-February-2018 | Landsat 7 ETM+ |
| 17-March-2018 | Landsat 8 OLI/TIRS | 2-April-2018 | Landsat 8 OLI/TIRS |
| 12-May-2018 | Landsat 7 ETM+ | 13-June-2018 | Landsat 7 ETM+ |
| 23-July-2018 | Landsat 8 OLI/TIRS | 16-August-2018 | Landsat 7 ETM+ |
| 9-September-2018 | Landsat 8 OLI/TIRS | 11-October-2018 | Landsat 8 OLI/TIRS |
| 28-November-2018 | Landsat 8 OLI/TIRS | 14-December-2018 | Landsat 8 OLI/TIRS |
| 7- January-2019 | Landsat 7 ETM+ | 24-February-2019 | Landsat 7 ETM+ |
| 28-March-2019 | Landsat 7 ETM+ | 5-April-2019 | Landsat 8 OLI/TIRS |
| 15-May-2019 | Landsat 7 ETM+ | 8-June-2019 | Landsat 8 OLI/TIRS |
| 18-July-2019 | Landsat 7 ETM+ | 27-August-2019 | Landsat 8 OLI/TIRS |
| 28-September-2019 | Landsat 8 OLI/TIRS | 30-October-2019 | Landsat 8 OLI/TIRS |
| 15-November-2019 | Landsat 8 OLI/TIRS | 17-December-2019 | Landsat 8 OLI/TIRS |
| Total: 240 scenes Landsat images (TM 45, ETM+ 126, OLI 69) | | | |
